# Benefit of Rare-Earth “Smart Doping” and Material Nanostructuring for the Next Generation of Er-Doped Fibers

**DOI:** 10.1186/s11671-017-1947-6

**Published:** 2017-03-21

**Authors:** Inna Savelii, Laurent Bigot, Bruno Capoen, Cedric Gonnet, Corinne Chanéac, Ekaterina Burova, Alain Pastouret, Hicham El-Hamzaoui, Mohamed Bouazaoui

**Affiliations:** 1 Univ. Lille, CNRS, UMR 8523, PhLAM - Physique des Lasers Atomes et Molécules, Lille, F-59000 France; 2 Prysmian Group, Z.I. Artois Flandres - Zone C, Billy Berclau, F-62092 France; 3 Sorbonne Universités - UPMC Univ Paris 06, CNRS, Collège de France, UMR 7574, Laboratoire de Chimie de la Matière Condensée de Paris, 4 place Jussieu, Paris Cedex 05, F-75252 France; 4 Present address: Saint-Gobain Recherche, 39 quai Lucien Lefranc, Aubervilliers Cedex, 93303 France

**Keywords:** Erbium-doped optical fiber amplifier, Oxide nanoparticles, Photoluminescence, Material nanostructuring

## Abstract

Erbium-doped fiber amplifiers (EDFAs) for harsh environments require to develop specific fabrication methods of Er ^3+^-doped fibers (EDFs) so as to limit the impact of radiation-induced absorption. In this context, a compromise has to be found between the concentration of Erbium and the glass composition. On the one hand, high concentration of Er ^3+^ ions helps to reduce the length of the EDF and hence the cumulated attenuation but generally leads to luminescence quenching mechanisms that limit the performances. On the other hand, so as to avoid such quenching effect, glass modifiers like Al ^3+^ or P ^5+^ ions are used in the fabrication of commercial EDFs but are not suitable for applications in harsh environment because these glass modifiers are precursors of radiation-induced structural defects and consequently of optical losses. In this work, we investigate the concept of smart doping via material nanostructuring as a way to fabricate more efficient optical devices. This approach aims at optimizing the glass composition of the fiber core in order to use the minimal content of glass modifiers needed to reach the suited level of performances for EDFA. Er ^3+^-doped alumina nanoparticles (NPs), as precursor of Er ^3+^ ions in the preform fabrication process, were used to control the environment of rare-earth ions and their optical properties. Structural and optical characterizations of NP-doped preforms and optical fibers drawn from such preforms demonstrate the interest of this approach for small concentrations of aluminum in comparison to similar glass compositions obtained by a conventional technique.

## Background

Since the mid-90s, EDFAs used in the context of long-haul transmission have proven their capacity to amplify efficiently and simultaneously several signal wavelengths. Today, they are deployed in very high data rate transmission systems with up-to-date modulation formats and detection techniques [1, 2]. Besides this success, some specific applications are still requiring improvement of the EDFA performances like high-power applications [3, 4] and use in harsh environments [5]. In the first case, it is highly desirable to mitigate the problem of concentration quenching that limits the performances of EDF presenting a doping level much higher than the one used for Telecom applications, i.e., few hundreds of ppm [6]. Indeed, it was shown that, at high erbium doping level, the distance between neighboring Er ^3+^ ions decreases, which increases the probability of Er-Er ions interaction. As a result, an energy transfer occurs between them, leading to the degradation of the performances of the amplifiers, especially the power conversion efficiency. Two mechanisms of energy transfer were identified depending on the distance between the neighboring Er ^3+^ ions: (i) homogeneous up-conversion when the doping ions are located at a nanometer scale distance from each other and (ii) the pair-induced quenching when the distance between the Er ^3+^ ions is shortened to an ion diameter scale. In the second case, an efficient energy transfer leads to the fact that the two ions can never be simultaneously excited to their first excited level (^4^I _13/2_ energy level). This ion-ion interaction is at the origin of a non-saturated absorption [7] and strongly depends on the manufacturing process [8]. Besides this concentration quenching effect, it was reported that EDFAs designed for space applications undergo a serious degradation of their performances under ionizing radiations [5, 9]. As an example, such an EDFA can present several decibels per meter of radiation-induced attenuation (RIA) in space environment where inter- or intra-satellite optical links are suited for space programs [10]. In this case, RIA leads to the degradation of the amplifier performances and hence of the transmission quality. It is known that glass modifiers ions (Al, P), inserted in the silica network so as to increase glass solubility or to improve the erbium spectral properties, induce structural defects in the fiber core and, in this way, increase the RIA [11]. It is particularly true for Al ^3+^ ions that are known to broaden and flatten the gain of EDFAs. The fabrication of fibers made of Al-free SiO_2_ doped with Er ^3+^ NPs solves this problem [12] but cannot be used for multi-wavelength channel application and are not compatible with high erbium concentrations. The two previous examples illustrate the need to master the local environment of erbium ions, which is not achievable by traditional doping techniques. In fact, by conventional methods, used for EDF fabrication, it is difficult to avoid the formation of Er ^3+^ ion pairs and the amount of glass modifiers must be maximized to reach the suited optical properties [13]. To this purpose, several approaches based on material nanostructuring are actively studied [14–16]. It is, for example, the case of a new doping technology that gives a supplementary degree of freedom on the design of active fibers and is fully compatible with the modified chemical vapor deposition (MCVD) manufacturing process [17–19]. It consists in the synthesis of Er ^3+^-doped NPs in solution that is thereafter used for a solution doping of optical fiber preforms, substituting a simple solution of Er ^3+^ salts. Thus, the rare-earth (RE) ions are dispersed in the NP structure, where a chemical control of the distance between neighboring Er ^3+^ ions, as well as of their close vicinity environment, is ensured. In consequence, material nanostructuring approach allows to reduce up-conversion losses and Er ^3+^ ion pair formation, especially for very high concentrations of RE ions [20]. Hence, this concept also presents a great interest for the development of EDFAs for space applications. In this paper, we will develop the concept of “smart doping” combined with material nanostructuring in order to improve the performances of the Er ^3+^-doped aluminosilicate optical fibers. The main idea consists in the minimization of glass modifiers content and the simultaneous maximization of the RE ions emitting capacity in the composition of the fiber core. We also target to highlight the effect of NP doping on structural and optical properties of EDFs. Alumina NPs have been chosen as a host for the Er ^3+^ ions due to their ability to incorporate a quite high level of RE ions in a highly dispersed state [21]. Hence, optical fibers drawn from Al_2_O_3_:Er ^3+^ NP-doped preforms with different aluminum contents have been fabricated. In order to estimate a competitiveness of Al_2_O_3_:Er ^3+^ NP-doped materials, the optical and lasing properties of Er ^3+^-doped optical fibers fabricated by NP-doping process are compared to those of standard EDFs.

## Methods

### Fiber Fabrication

The first step of the optical fiber manufacturing consisted in the synthesis of Al_2_O_3_:Er ^3+^ NP precursor by the co-precipitation of Er ^3+^ and Al ^3+^ salts. This process was previously described in references [17, 18]. Thus, Er ^3+^-doped alumina NPs with different Al/Er molar ratios (30, 50, and 200) have been fabricated in order to find the optimum ratio for the targeted optical performances [9]. Regarding the fiber fabrication, a Germanium-doped porous silica layer was deposited by the MCVD method on the inner surface of a substrate tube (outer diameter 30 mm, inner diameter 26 mm) at a temperature about 1400 °C. Then, this soot layer was soaked in the suspension of doped NPs. The solution was then removed, and after dehydration (2 h at 1000 °C), collapsing (at 2000–2200 °C) and sleeving, the fabricated preform was drawn into fiber. The standard manufacturing of Er ^3+^-doped fibers followed the same fabrication steps, except that inorganic salts of Al ^3+^ and Er ^3+^ ions were used instead of the NP solution. Optical fibers drawn from Al_2_O_3_:Er ^3+^ NP-doped preforms have been fabricated with Al ^3+^ mass content varying from 0.35 to 4.5 wt%. These fibers were compared to the conventional fibers with usual Al ^3+^mass content (4.0 and 6.0 wt%) and with low Al ^3+^content (0.35 wt%) (see Table 1). So as to reach the same opto-geometrical characteristics, the refractive index of the fabricated fibers was adjusted by germanium content in the fiber core glass composition. The Er ^3+^ concentration in the fabricated samples varied from 160 to 470 wt ppm with an increase of Al ^3+^ concentration.

**Table 1:** Fiber core glass compositions (wt%), Al/Er ratio (mol%), numerical aperture (NA), and core diameter (*μ*m)

Sample reference	Process type	Al content	Ge content	Al/Er	NA	Core diameter
NP035	Nano	0.35	23	30	≃0.27	≃ 4
NP100	Nano	1.00	3	50	≃0.2	idem
NP180	Nano	1.80	5	30	idem	idem
NP350	Nano	3.50	3	200	idem	idem
NP450	Nano	4.50	0.75	200	idem	idem
ST035	Standard	0.35	25	57	≃0.27	idem
ST400	Standard	4.00	<0.5	444	≃0.2	idem
ST600	Standard	6.00	<0.5	800	≃0.27	idem

### Characterization

Morphology of the NP precursor and of the NPs in the preform core was studied by transmission electron microscopy (TEM), performed with a microscope FEI Tecnai G2-20 Twin using a 200-kV acceleration voltage. For the sample preparation, either a suspension of NP precursor or a piece of preform core grinded into a fine powder was directly deposited onto a 200-mesh Cu grid previously coated with a thin carbon membrane. These samples were then metalized with a vaporized carbon layer. Doped NPs were analyzed by powder X-ray diffraction (PW-XRD) using a Bruker D8 Advance diffractometer using Cu-K radiation (*λ* = 1.5418 Å) at 45 kV and 40 mA, 0.0046 ^∘^ 2 *θ* step size, and 0.5 s step time over a range of 10 to 80° 2 *θ*. The composition of the core of the preform was determined by electron probe microanalysis (EPMA). The emission spectra of the fabricated fibers were recorded at room temperature with a Horiba Jobin-Yvon IHR550 spectrometer coupled with a nitrogen-cooled multi-channel InGaAs detector. The near infrared (NIR) photoluminescence (PL) was collected transversely a few centimeters after the injection point, using a continuous-wave 980 nm emitting laser diode as the excitation source. Background optical losses were measured by the cutback method with an optical fiber analyzer (PK2210 from Photon Kinetics). Typically, fiber lengths of about 100 and 2 m were used to record the two transmission curves. Er ^3+^-related absorption bands were recorded using a white light pump source together with an optical spectrum analyzer. The fiber under test (less than 5 m in length) was spliced at both ends to single-mode pigtails. Laser properties were characterized in an all-fiber laser configuration [22]. A 974-nm pigtailed laser diode was spliced to a high-reflectivity fiber Bragg grating (FBG) with a wavelength centered at 1530 nm. This fiber was then spliced to the Er ^3+^-doped fiber sample under test, and the laser cavity was hence constituted by the FBG and a cleaved end facet. The output beam was measured with a powermeter. Note that a long-pass filter was used to stop the residual pump beam. The experimental setup was calibrated in order to determine the losses induced by lens and filter. It has to be noticed that fibers compared in laser configuration were designed in order to have the same opto-geometrical characteristics: numerical aperture in the 0.2–0.27 range, mode field diameter of about 4 *μ*m, and cut-off wavelength in the 1130–1140-nm wavelength range. Non-saturable absorption as a function of optical power was characterized for the fiber equivalent length of about 32-dB small signal absorption. A tunable laser source was used to adjust the probe wavelength to the fiber absorption peak wavelength (1531.2 nm). The probe beam was amplified by an EDFA and then varied by use of a variable optical attenuator. A tunable band-pass filter was used to remove the amplified spontaneous emission (ASE) generated in the EDFA. The transmission curves of the EDFs were recorded as a function of the input power (Pin) varying from about −30 to 20 dBm. The output power (Pout) and the corresponding transmission value were corrected from splice losses and from characteristics of wideband coupler placed just before the EDF in the experimental setup [23].

## Results and Discussion

### Structural Characterization

**Fig. 1:**
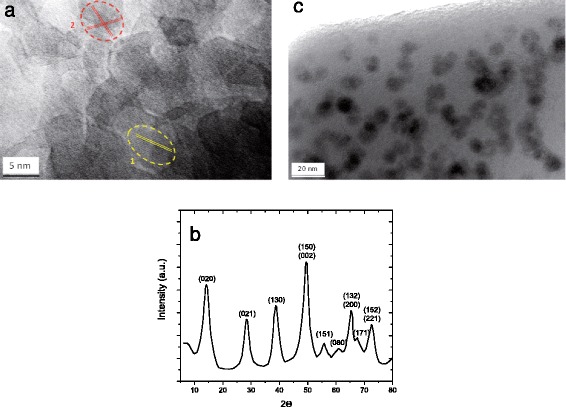
**a** TEM image of Er ^3+^ -doped boehmite NPs in the precursor solution. **b** XRD pattern of Er ^3+^-doped boehmite with Al/Er = 200. **c** TEM image of Al_2_O_3_:Er ^3+^ NPs embedded in the optical fiber preform core of the sample NP350

The investigation of the influence of the NP-doping process on the material properties started from the structural characterizations of the NP-doped solution and of the corresponding NP-doped preforms. Hereafter, one example of such a study is detailed for the fabrication of Al_2_O_3_:Er ^3+^ NP-doped fiber NP350. In a previous work, it has been shown that NP morphology depends on the material crystalline structure [24] and that NP size is a function of synthesis conditions (temperature, pH, aging time...) [25]. Thus, in the present conditions, namely after the co-precipitation of Al ^3+^ and Er ^3+^ ions at pH = 9, Er ^3+^-doped boehmite AlOOH NPs with a mean size of 5 nm are found, as is shown in Fig. 1 a that presents a HR-TEM image of the NP precursor in solution. The interplanar distances have been analyzed in order to determine the NP structure. Two circled zones present the general structural trend of NP precursor. The interplanar distance of the first zone is 2.4 ± 0.1 Å. The second zone presents a superposition of two NPs with interplanar distances of 2.38 ± 0.17 Å and 2.35 ± 0.08 Å. These values can be attributed to the (130) lattice planes of boehmite AlOOH crystal structure (d130 = 2.35 Å) [26]. XRD pattern of doped NPs is given in Fig. 1 b together with peak attribution. The mean particle size deduced from the broadening of diffraction peaks is in good agreement with TEM analysis, thus confirming that particles are mono-crystalline. The small size of Er ^3+^-doped boehmite NPs in the precursor solution makes them fully compatible with a liquid-doping step in the fabrication process where the porosity of the MCVD-made soot layer is larger than several micrometers [27]. Hence, the Er ^3+^-doped boehmite NP solution substitutes to the simple solution of Er ^3+^ ions used in the conventional preform soaking step. Figure 1 c presents the TEM image of Al_2_O_3_:Er ^3+^ NPs in the optical fiber preform sample NP350. Heterogeneous and apparently amorphous structures are observed in contrast to the homogeneous glass matrix of standard Er ^3+^-doped preforms (also analyzed but not shown here). Dark zones are attributed to the alumina NPs with a mean size of 10 nm. It is thus concluded that, after the high-temperature densification of the preform, AlOOH NPs are transformed into the more thermodynamically stable gamma phase of alumina NPs [17]. The size of alumina particles is around 10 nm, suggesting the sintering of two or three boehmite particles only.

### Optical and Spectroscopic Properties

**Fig. 2:**
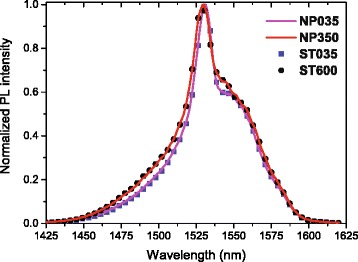
Room temperature normalized PL spectra

In order to investigate the influence of NP-doping process on the spectroscopic properties of Er ^3+^-doped fibers, PL properties of the fabricated fibers (Table 1) have been studied. If crystalline Al_2_O_3_ NPs would have been obtained, Er ^3+^ ions would be surrounded by periodic arrangement of Al ^3+^ ions, the crystal field of which results in a structured emission spectrum of the erbium ions. However, in preforms and optical fibers, as mentioned in reference [17], the host glass matrix also impacts on the nature of Er ^3+^ ion environment. Moreover, the alumina particles embedded in the silica matrix seem to be amorphous. Indeed, as shown on Fig. 2, broad PL spectra of optical fibers drawn from Al_2_O_3_:Er ^3+^ NP-doped preforms with low and high concentrations of Al ^3+^ ions suggest the amorphous environment of Er ^3+^ ions, as it is the case for standard EDFs. This is consistent with the rapidity of the heat treatment involved in the fiber-drawing process and also with the small dimensions of these nanoparticles (< 15 nm) [28]. It can also be seen on Fig. 2 that normalized NIR PL spectra of Er ^3+^-doped fibers fabricated by standard and by the NP-doping processes with the lowest Al ^3+^ ions content (NP035 and ST035) have similar shape. In both doping processes (standard Al-doping or Al_2_O_3_:Er ^3+^ NP-doping), an increase in Al ^3+^ ion concentration broadens progressively the PL spectra, mostly through the shoulder around 1500 nm. It is observed that FWHM increases with Al ^3+^ ions content owing to the change in inhomogeneous and homogenous broadenings, as well as to the modification of the Stark splitting of excited and ground states. Peak position is also a probe of the Er ^3+^ ion close vicinity environment. In general, Er ^3+^-doped aluminosilicate fibers with high amount of Al ^3+^ ions have their main emission peak centered near 1529 nm, which is also the case here [29]. This blue shift of the main emission band, together with the increase of the FWHM, indicates that Er ^3+^ ions in the optical fibers drawn from Al_2_O_3_:Er ^3+^ NP-doped preforms have Al ^3+^ ions in their vicinity and behave similar to standard fibers. It has to be pointed out that luminescence decay—not shown here—are also very similar to samples obtained by both methods and are about 10 ms. Thus, application of “smart doping” concept, combined with material nanostructuring, allows to fabricate Er ^3+^-doped fiber without degradation of PL shape and dynamics. Hence, the environment of RE ion controlled by its NP host matrix composition will also ensure the standard gain bandwidth and the targeted amplification properties [8, 17–20].

**Fig. 3:**
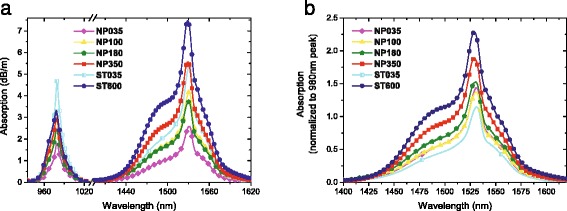
**a** NIR absorption spectra and **b** NIR absorption spectra of Er ^3+^-doped fibers normalized to the absorption peak around 980 nm and centered around 1530 nm

Taking this into consideration, further investigation of the optical and laser properties was achieved for the optical fibers drawn from Al_2_O_3_:Er ^3+^ NP-doped preforms with the Al ^3+^ ion concentration from 0.35 to 3.5 wt%, which were compared to the properties of the standard fibers with low (0.35 wt%) and high (6 wt%) Al ^3+^ ion content. Erbium-related absorption and background optical losses were measured by the cutback method. All the fabricated fibers exhibit background attenuation losses below 3 dB/km at 1150 nm. OH content was estimated below 1 wt ppm. This suggests that the AlOOH:Er ^3+^ NPs have been transformed into the Al_2_O_3_:Er ^3+^ NPs before the densification step of RE NP-doped preform fabrication. Figure 3 a presents Er ^3+^-related NIR absorption spectra of fabricated fibers in the 935–1630-nm wavelength range. In Fig. 3 b, these spectra have been normalized to their peak at 978 nm. This ratio illustrates the absorption properties at 1529 nm as a function of absorption properties around 980 nm. As can be deduced from Fig. 3 b, the ratio between the absorption around 1530 nm and the absorption around 980 nm (1530/980) varies from one fiber to the other. In the case of the fibers with 0.35 wt% Al ^3+^, the ratio is larger for the optical fibers drawn from Al_2_O_3_:Er ^3+^ NP-doped preforms than for the standard fiber (1.43 instead of 1.13). Comparing the opto-geometrical parameters of these two fibers and hence the overlap integral between the guided mode and the doped region, it could be concluded that the guiding properties of the two fibers are the same. Consequently, it means that NP-doping process modifies the ratio between the cross sections of the two main absorption bands. It can be deduced that, for this Al ^3+^ content, either the absorption cross section at 1530 nm is increased or absorption cross section at 980 nm is decreased when using the NP precursor. In the case of NP-doped fibers containing moderate concentrations of Er ^3+^ and Al ^3+^ ions and achieved using more or less the same Al/Er ratio (30 and 50), similar absorption properties were observed (fibers NP035, NP100, and NP180).

### Laser Properties

**Fig. 4:**
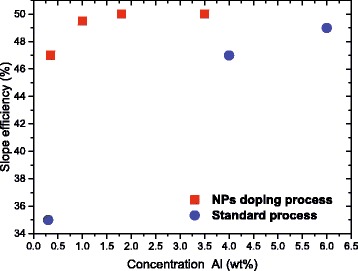
Slope efficiency of optical fibers drawn from Al_2_O_3_:Er ^3+^ NP-doped preforms as a function of Al content

**Fig. 5:**
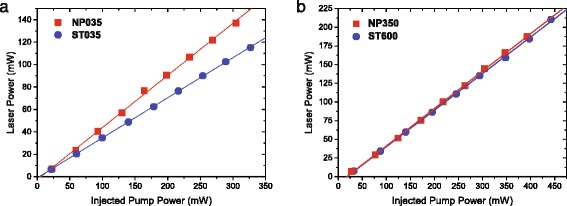
Output power of laser emission at 1530 nm versus pump power for the all-fiber Er ^3+^-doped fiber laser. **a** Low Al content. **b** High Al content

In order to estimate the potential of NP-doped materials for amplifier application, we performed a characterization of the lasing properties of the optical fibers drawn from Al_2_O_3_:Er ^3+^ NP-doped preforms and the Er ^3+^-doped standard optical fibers with low (0.35 wt%) and high (3.5 and 6 wt.%) aluminum contents. In both cases, the output laser power at 1530 nm was measured as a function of the injected pump power. The optimal fiber lengths were experimentally determined by studying the slope efficiency and output laser power as a function of the fiber length. The maximum of slope efficiency has been observed for the values corresponding to about 30 dB of small-signal absorption at the pump wavelength. Figure 4 illustrates the correlation between the slope efficiency and Al ^3+^ content of the optical fibers drawn from Al_2_O_3_:Er ^3+^ NP-doped preforms. A correlation exists between laser efficiency and aluminum content, small aluminum content being associated to reduced efficiency. Moreover, it is observed that, for 0.35 wt% Al ^3+^ ions, the slope efficiency of the optical fibers drawn from Al_2_O_3_:Er ^3+^ NP-doped preforms is about 47% whereas efficiency of Er ^3+^-doped standard optical fiber is only about 35% (Fig. 5 a). Note that a similar trend (not shown here) has been observed using 4–4 % laser cavities for both fibers. A further increase of the Al ^3+^concentration (up to 1.8 wt%) allows to reach the slope efficiency of 50%, similar to the one measured for standard fiber with Al ^3+^ content of 6 wt% (Fig. 5 b). In these experiments, the maximum output power was only limited by the available pump power. Indeed, NP-doping process allows to reduce the Al ^3+^ ion concentration while preserving laser properties similar to the ones of a more doped fiber whereas, at lower concentration, it helps to maintain a quite high level of laser performances with a reduced aluminum content.

### Non-saturable Absorption

**Fig. 6:**
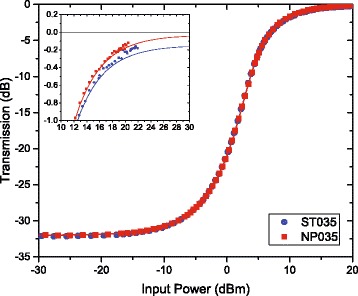
Non-saturable absorption measurements of optical fibers drawn from Al_2_O_3_:Er ^3+^ NP-doped preforms NP035 and Er ^3+^-doped standard optical fiber ST035. *Inset*: zoom at high input power. *Lines* are guides for the eye

In general, the concentration quenching is at the origin of EDFA performance degradation. Applying of NP-doping concept should help to reduce the pair formation inside the NPs, but it does not prevent the potential aggregation of the NPs in the doping solution [8, 17–20]. Hence, the measurement of non-saturable absorption was performed in order to understand the difference in the laser properties of the fibers with a low Al ^3+^ ion content fabricated by standard and NP-doping processes. Figure 6 presents their transmission curves as a function of input power. Curves from both Er ^3+^-doped fibers demonstrate a similar shape. A plateau is observed for the high input power values and allows to quantify the number of Er ^3+^-Er ^3+^ pairs. The value of this plateau, NSA (in dB), is given by the following analytic equation extracted from reference [6] and permits to determine the fraction of ion pairs:

1 $$ \text{NSA}=-\frac{10}{\text{ln}10}\sigma_{a}\Gamma N_{Er} L_{\text{fiber}} m \left(1-\frac{\sigma_{a}+\sigma_{e}}{2\sigma_{a}+\sigma_{e}}\right)   $$

where *σ*_*a*_ and *σ*_*e*_ are the absorption and emission cross sections, respectively, *Γ* is the mode-dopant overlap integral, *N*_*Er*_ is the erbium concentration, *L*_fiber_ is the fiber length, and *m* is the fraction of pair. Thus, it is estimated that optical fibers drawn from Al_2_O_3_:Er ^3+^ NP-doped preforms contain about 0.2% Er-Er pairs whereas Er ^3+^-doped standard fiber has a percentage of pairs equal to about 1.2%. This result is comparable to the previous investigation of pair formation in the fibers fabricated by the same route [20]. The difference of pair percentage between the fabricated fibers combined to the modification of 1530/980 absorption ratio partly explains the difference in laser efficiencies reported on Fig. 5 a between fibers NP035 and ST035. For a similar glass composition and fiber geometry, NP-doping process helps to take better benefit of erbium properties when compared to a standard process.

## Conclusions

In this article, the influence of the nature on the erbium precursor for the synthesis of Er ^3+^-doped aluminosilicate optical fibers has been investigated, applying the concepts of “smart doping” and material doping nanostructuration. Crystalline Al_2_O_3_:Er ^3+^ NPs have been synthesized and then used for the solution doping of optical fiber preforms. TEM images permitted to identify Al_2_O_3_:Er ^3+^ NPs in the core of the preforms. Optical and spectroscopic properties of such optical fibers drawn from Al_2_O_3_:Er ^3+^ NP-doped preforms have been presented together with a demonstration of their lasing properties at 1530 nm. All these properties have been compared to those obtained for fibers made by a conventional process. For a similar Al content, optical fibers drawn from Al_2_O_3_:Er ^3+^ NP-doped preforms are more efficient than standard fibers at low Al concentration and show similar performances at higher Al content. Modification of the intensity of emission and absorption cross sections, together with a reduced percentage of erbium pairs, partly explain these results. We believe that this fabrication technique, by minimizing the concentration of glass modifiers, could benefit to EDFAs developed for space applications and, more generally, to EDFAs for which a high power conversion efficiency is required [30]. It also opens some interesting perspectives on the investigation of more complex glass composition suitable for high-power applications [31].
